# Receiver operating characteristic analysis to determine optimal cutting point of cage in predicting physical and mental comorbidities among alcohol users

**DOI:** 10.1192/j.eurpsy.2021.1494

**Published:** 2021-08-13

**Authors:** C.Y. Chu, S.C. Wang, C.H. Lee, C.M. Cheng

**Affiliations:** 1 Department Of Forensic And Addiction Psychiatry, Jianan Psychiatric Center, Ministry of Health and Welfare, Tainan, Taiwan; 2 Consultant Physician, Jianan Psychiatric Center, Ministry of Health and Welfare, Tainan, Taiwan

**Keywords:** receiver operator characteristic (ROC) analysis, alcohol use disorder (AUD), comorbidities, CAGE

## Abstract

**Introduction:**

Alcohol use disorder (AUD) is highly related to various comorbidities, such as cancer, cognitive impairment, cirrhosis, chronic sclerosing stomatitis, stroke, and depression. The CAGE (Cut down, Annoyed, Guilty, Eye-opener) questionnaire is a simple screening material to make a diagnosis of alcoholism.

**Objectives:**

Our study aimed to find an optimal cut-off point of CAGE for alcohol-related comorbidities in Taiwan.

**Methods:**

We performed demographic analysis for 280 participants with AUD and categorized them into two groups according to CAGE scores. We applied receiver operator characteristic (ROC) analysis to determine optimal cutting point of CAGE in predicting physical and mental problems among alcohol users. Statistical analysis was performed with the Statistical Software Stata version 12.0 (StataCorp LP, College Station, TX, USA).

**Results:**

The mean age of participants was 45.9 ± 10.5 years, and all of them were male. Among 280 participants, 134 (47.9%) had physical diseases, including 37 (13.2%) with liver disease, 10 (3.6%) with pancreatitis, 22 (7.9%) with gout, and 5 (1.8%) with esophageal varices; while 33 (11.8%) had one or more mental illnesses. Patients with CAGE score greater than 3 were more likely to have both mental health problems and/or physical diseases, especially hepatic disease and esophageal varices.

**Figure:**
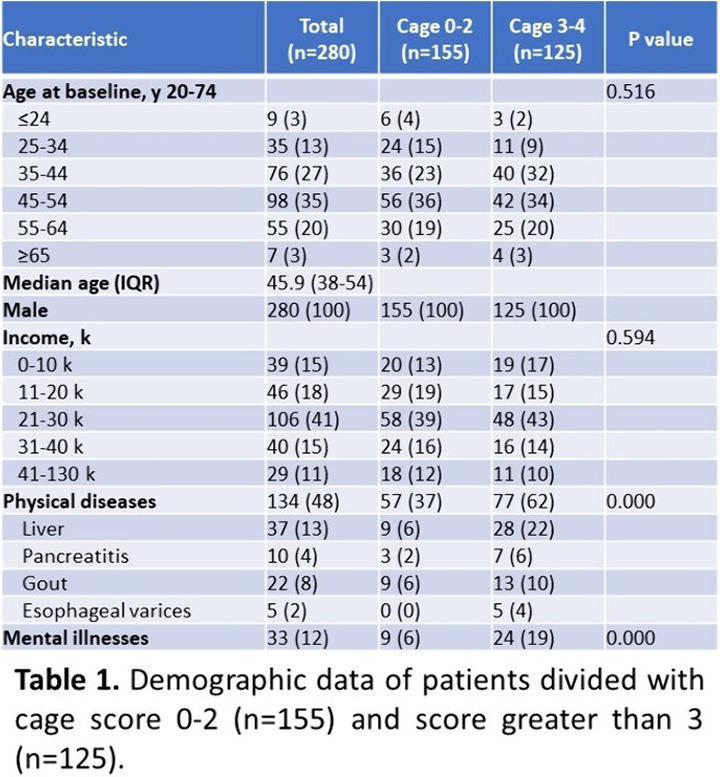

**Figure:**
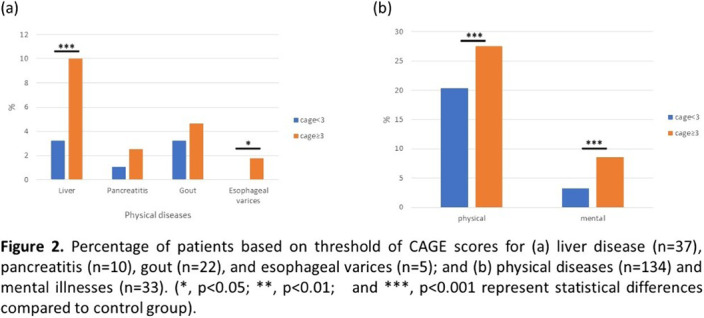

**Conclusions:**

This study revealed that with CAGE score greater than 3, male patients with AUD are at higher risks of both physical and mental comorbidities. Further research as well as female participants are needed to identify the associations between the severity of alcohol use disorder and related diseases for comprehensive evaluation in Taiwan.

